# Risk factors of oncogenic HPV infection in HIV-positive men with anal condyloma acuminata in Shenzhen, Southeast China: a retrospective cohort study

**DOI:** 10.3389/fpubh.2023.943115

**Published:** 2023-12-11

**Authors:** Jiaxin Liu, Rongqing Yang, Xiaobao Zhao, Wenzhu Chu, Dapeng Li, Fuxiang Wang, Lanlan Wei

**Affiliations:** ^1^National Clinical Research Center for Infectious Diseases, Institute for Hepatology, The Third People's Hospital of Shenzhen, The Second Hospital Affiliated to Southern University of Science and Technology, Shenzhen, China; ^2^School of Medicine, Taizhou Polytechnic College, Taizhou, China; ^3^Department of Dermatovenerology, The Third People's Hospital of Shenzhen, The Second Hospital Affiliated to Southern University of Science and Technology, Shenzhen, China; ^4^Department of Dermatology, Hongqi Hospital, Mudanjiang Medical University, Heilongjiang, China; ^5^Department of Infectious Diseases, The Third People's Hospital of Shenzhen, Second Hospital Affiliated to Southern University of Science and Technology, Shenzhen, China

**Keywords:** HIV, HPV, oncogenic HPV infection, condyloma acuminata, anal cancer, risk factors

## Abstract

**Background:**

Human immunodeficiency virus (HIV)-positive patients with anal condyloma acuminata (CA) present an increased risk of anal cancer progression associated with oncogenic human papillomavirus (HPV) infection. It is essential to explore determinants of anal infection by oncogenic HPV among HIV-positive patients with CA.

**Methods:**

A retrospective cohort study was performed in HIV-positive patients with CA between January 2019 to October 2021 in Shenzhen, Southeast China. Exfoliated cells were collected from CA lesions and the anal canal of HPV genotypes detected by fluorescence PCR. Unconditional logistic regression analysis was used to probe associations of independent variables with oncogenic HPV infection.

**Results:**

Among HIV-positive patients with CA, the most prevalent oncogenic genotypes were HPV52 (29.43%), HPV16 (28.93%), HPV59 (19.20%), and HPV18 (15.96%). Risk of oncogenic HPV infection increased with age at enrollment (COR: 1.04, 95% CI: 1.01–1.07, *p* = 0.022). In the multivariable analysis, age ≥ 35 years (AOR: 2.56, 95% CI: 1.20–5.70, *p* = 0.02) and history of syphilis (AOR: 3.46, 95% CI: 1.90–6.79, *p* < 0.01) were independent risk factors statistically associated with oncogenic HPV infection. History of syphilis (AOR: 1.72, 95% CI: 1.08–2.73, *p* < 0.02) was also an independent risk factor statistically associated with HPV16 or HPV18 infection.

**Conclusion:**

In clinical practice, HIV-positive CA patients aged ≥35 years or with a history of syphilis should carry out HR-HPV testing and even anal cancer-related examinations to prevent the occurrence of anal cancer.

## 1 Introduction

The human immunodeficiency virus (HIV) epidemic is predominant among men who have sex with men (MSM) (1). The overall prevalence of HIV among the Chinese MSM population displays an increased tendency over time (2). Among several common morbidities caused by HIV infection (3), condyloma acuminata (CA) and anal ulcers account for the vast majority of anal diagnoses (4). Since the recurrence of anal CA in HIV-positive patients is common and frequently accompanied by human papillomavirus (HPV) infection (5) and occult tumorigenesis (6), HIV-positive patients with anal CA have received increasing attention in recent years.

Anal HPV infection is almost universal in HIV-positive MSM (7). HPV is considered the most common sexually transmitted infectious agent (8). Intraepithelial infection of the anal canal with different genotypes of HPV may develop histologically distinct types of lesions (9). Over 120 types of HPV are categorized further as high-risk HPV (HR-HPV) and low-risk HPV (LR-HPV) based on their carcinogenic potential (10). HR-HPV infection is strongly associated with high-grade anal intraepithelial neoplasia and anal cancer (11, 12). LR-HPV infection, especially LR-HPV 6 or 11, contributes to 90% of the cases of CA, which are traditionally considered benign lesions (13), which makes it seem that patients with anal CA do not need special concern. However, HIV-induced immunodeficiency has complicated effects on CA and HPV infection. Patients with HIV tend to develop larger or more warts and have a higher co-infection rate of HR-HPV that causes anal cancer, particularly HPV-16 (14). Moreover, anal intraepithelial neoplasia can be hidden in condylomas (15). A higher percentage of high-grade dysplasia or even invasive squamous cell carcinoma is found in condylomas of HIV-positive MSM compared with HIV-negative MSM (6, 13, 16). Therefore, HIV-positive patients with anal CA present an increased risk of anal cancer progression (17).

Anal cancer is an increasingly common cancer among the HIV-infected population (18). Reports have suggested that the risk of anal cancer in HIV-positive patients is not reduced by highly active antiretroviral therapy (HAART) (19–21). Although HPV vaccines are recognized as effective in reducing anal intraepithelial neoplasia in HIV-positive patients (18), HPV-associated anal cancer remains a considerable burden due to the high cost of vaccination. More importantly, no consensus screening guidelines exist for anal cancer (22). A prospective study among the HIV-positive MSM population reported that abnormal cytology and oncogenic HPV determination showed similar sensitivity in detecting high-grade anal intraepithelial neoplasia (23). From this, avoiding oncogenic HPV infection may be a feasible strategy for preventing anal cancer in HIV-positive CA patients.

Currently, anal cancer screening procedures are not routinely performed in HIV-positive patients with CA in China, which leads to neglected cancer risk. This study aimed to assess determinants of oncogenic HPV infection in HIV-positive patients with CA, hoping to provide a practical reference for the screening strategy of anal cancer.

## 2 Materials and methods

### 2.1 Study design and data collection

A retrospective cohort study was performed in the Department of Dermatology and Venereology of Shenzhen Third People’s Hospital from January 2019 to October 2021. The design and study protocol was approved by the independent ethics committee of the hospital, and informed consent was obtained from all the patients. HIV-positive patients with CA who underwent anal HPV infection and genotyping test as part of their routine auxiliary examination were enrolled in the cohort. Patients were excluded if they had a history of (or current) anal intraepithelial neoplasia, anal cancer, verrucous cancer, CA with atypical hyperplasia, or other benign lesions.

For eligible participants, HPV, syphilis, HIV infection detection, and HIV-related parameters were performed at the same time as the diagnosis of CA. If participants had a history of syphilis or HIV infection, the physician obtained clinical characteristic data by asking for medical history and reviewing electronic medical records. The clinical characteristic data involved include demographic characteristics (age and marital status), STI history, sexual behavior, orientation, time of initial diagnosis of HIV or CA (duration of HIV or CA), history of CA or anti-HIV treatment, and HIV-related parameters.

### 2.2 Diagnostic criteria and clinical tests

CA and HIV infections were diagnosed by experienced doctors according to respective country guidelines. According to the Guideline for the Clinical Management of Anogenital Warts in China (24), CA was diagnosed clinically by a history of exposure combined with typical skin lesions (e.g., cauliflower-like masses), dominated by visual and physical examination and sometimes aided by an anoscopy and proctosigmoidoscopy (25). In contrast, atypical skin lesions were confirmed by pathological biopsy. The treponema pallidum particle agglutination assay (TPPA) was performed on patients with CA at the time of their initial visit to our hospital. If the TPPA test was positive, the patient was considered to have a history of syphilis.

The HIV-1/2 antibody test was the gold standard for diagnosis of HIV infection, which was conducted using ELISA in this study. However, according to the Chinese guidelines for the diagnosis and treatment of HIV/AIDS (2021 edition) and the National Guideline for Detection of HIV/AIDS (2022), if the preliminary screening found positive HIV-1/2 antibody, the local CDC must repeat the test for final confirmation (26).

CD4^+^ cell count and TH/TS ratio were detected by flow cytometry (BD Biosciences, United States), and HIV RNA quantitative detection was performed using PCR with HIV-1 RNA Quantitative Diagnostic Kit (DAAN GENE, Guangzhou, China).

### 2.3 Specimen collection and HPV genotyping

To optimize HPV analysis accuracy, patients were required to refrain from washing the anal canal area for several hours, have no anal sex in 24 h, and not use lubricants and medications before sampling.

The collection of specimens was performed by dermatologists with relevant experience. Exfoliated cells were obtained by wiping CA lesions and the anal canal with a dedicated swab (Jianyou Medical Technology, Jiangsu, China) 3–5 times and flushed from the swab into 2 mL of phosphate-buffered saline. The collected specimens were stored at 4°C, and DNA extraction and HPV genotyping were performed within 24 h.

DNA was extracted by a DNA Extraction (boiling method) Kit (BioPerfectus Technologies, Jiangsu, China) according to the manufacturer’s instructions. In total, 500 μL of exfoliated cell samples were taken and centrifuged at 12000 rpm for 5 min. The supernatant was discarded, and 100 μL of lysate was added. After 10 min of dry bath, the mixture was blended well and centrifuged at 12000 rpm for 5 min, then the supernatant (nucleic acid) was retained and stored at −20°C.

HPV genotyping was performed using fluorescence PCR with a Human Papillomavirus Nucleic Acid Typing Kit (BioPerfectus Technologies, Jiangsu, China) for 21 HPV types, which contain 13 HR-HPVs: HPV-16, HPV-18, HPV-31, HPV-33, HPV-35, HPV-39, HPV-45, HPV-51, HPV-52, HPV-56, HPV-58, HPV-59, and HPV-68. A total of 21 type-specific primers and probes were designed to target the L1 region of HPV. The fluorescent PCR instrument (Hongshi Medical Technology Co., LTD, Shanghai, China) could automatically draw a real-time amplification curve according to the fluorescence signal to carry out qualitative detection of HPV DNA. A total of 2 μL of DNA templates was required and the total reaction volume was 20 μL. PCR reactions were conducted according to the following cycling conditions: incubation with uracil DNA glycosylase at 50°C for 5 min; initial denaturation at 95°C for 10 min; 45 cycles of 95°C for 10 s and 58°C for 40 s. Perfectus software v1.0 (Bioperfectus Limited Corp., China) was used for genotyping analysis. The HPV type was considered positive if the corresponding amplification curve showed a typical S-shape and the CT value was less than the reference. The sensitivity, specificity, and accuracy of the HPV assay are 98.4%, 99.6%, and 99.6%, respectively (27), and it has also been used by other researchers (28, 29).

### 2.4 Study variables and statistical analysis

Oncogenic HPV infection was defined as infection with at least one of the 13 HR-HPV types, regardless of the presence or absence of co-infection with LR-HPV types. Participants who reported being widowed and divorced were classified as unmarried. The duration and therapy of CA were based on the current visit. The history of CA, which had been cured in the past, was not included in the study. The partitioning of HIV-related categorical variables was determined based on actual reference values in clinical practice.

All analyses were performed in R software [R version 4.1.1 (2021-08-10)— “Kick Things” Copyright (C) 2021, The R Foundation for Statistical Computing Platform: x86_64-w64-mingw32/x64 (64-bit)]. The baseline characteristics of the study population were analyzed and presented as frequencies and median (IQR). Chi-squared or Fisher’s exact tests were performed to compare differences in clinical and socio-demographic characteristics among oncogenic HPV-positive and oncogenic HPV-negative participants. Odds ratios (ORs) were reported with 95% confidence intervals (CIs). Unconditional logistic regression analysis was used to probe associations of independent variables with oncogenic HPV infection. *p*-values of <0.05 were considered statistically significant.

## 3 Results

### 3.1 Participant screening

**Figure 1:**
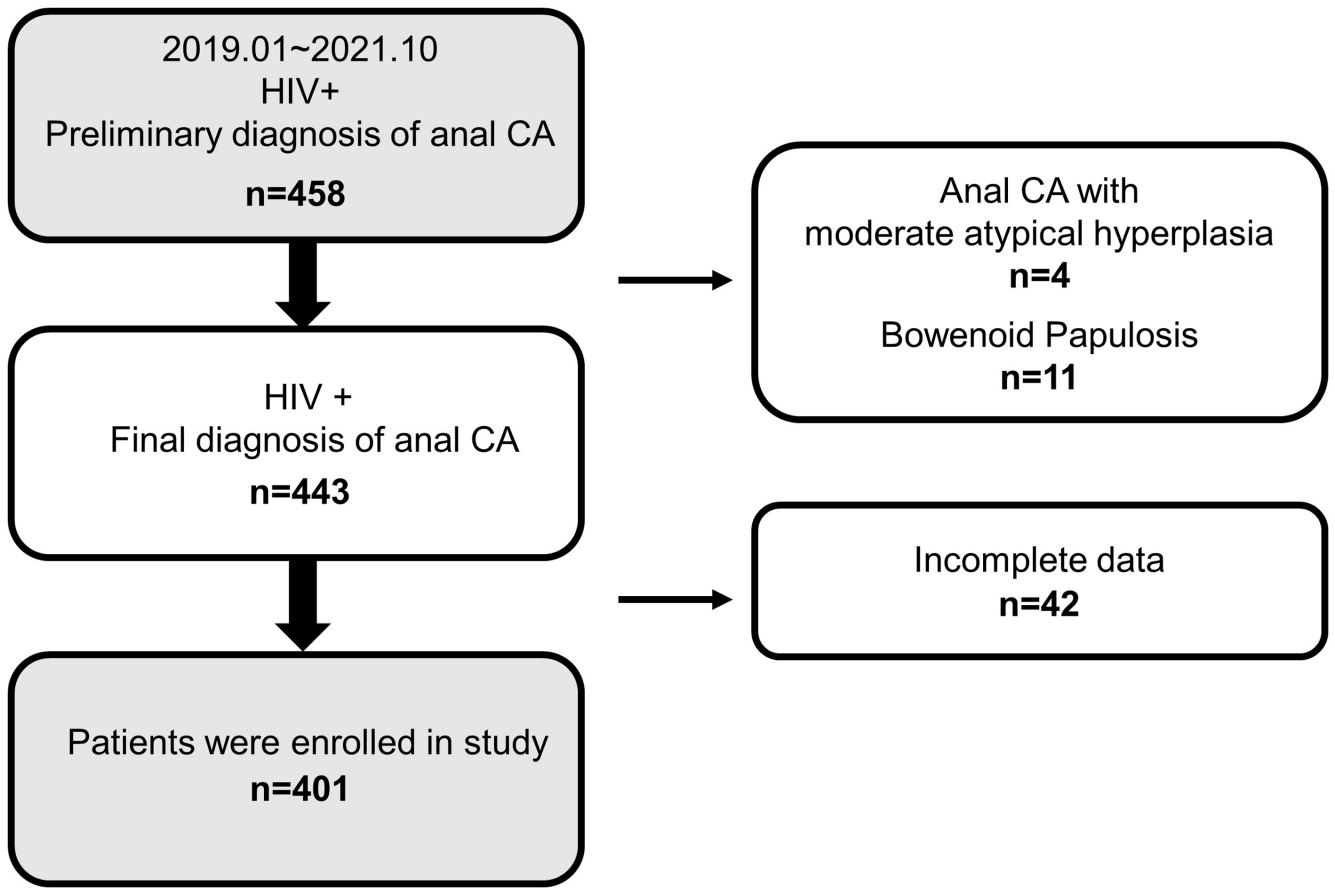
Overview of exclusions and final study population. CA, condyloma acuminata; HIV+, HIV positive; −, HIV negative.

The cohort comprised 401 HIV-positive men who were suffering from anal CA. A flowchart of the screening participants is shown in Figure 1. A total of 458 HIV-positive patients with a preliminary diagnosis of anal CA were included from January 2019 to October 2021, of which 15 patients had malignant-prone lesions (4 CA patients with moderate atypical hyperplasia and 11 bowenoid papulosis) were excluded. Subsequently, among the 443 patients with a final diagnosis of anal CA, 42 patients were excluded due to incomplete data, resulting in 401 participants. It should be noted that female patients were naturally excluded from the cohort without gender restrictions on study subjects. Consequently, the subjects of this study were all HIV-positive men with anal CA.

### 3.2 Participant characteristics

During the study period, the characteristics of participants are shown in Table 1. The cohort of 401 HIV-positive men had a mean age of 31 years (SD, 8.58; range, 18–72 years), their median age was 29 years, and most participants were under 35 (73.32%). Overall, nearly one-third of participants were unmarried (26.93%); the vast majority of the participants were MSM (95.01%) and had anal sex (94.51%), and only 29.93% had a history of syphilis. In addition, 284 participants (70.82%) had a duration of CA of less than 1 year, and 274 participants (68.33%) were not treated. A large proportion of participants had a duration of known HIV infection (67.58%) or HAART (71.82%) less than 3 years, 23.69% had an HIV-RNA ≥ 500 IU/mL, 67.83% had a TH/TS ratio of <0.6, and only 13.47% had a CD4^+^ count <200 cells/μl.

**Table 1:** Clinical and socio-demographic characteristics of the study population.

Characteristics	Total (*N* = 401)
	*n* (%)
Age group (year)	
≤ 26	93 (23.19)
27–30	109 (27.18)
31–34	92 (22.94)
≥ 35	107 (26.68)
Marriage	
Yes	293 (73.07)
No	108 (26.93)
Men had sex with men	
Yes	381 (95.01)
No	20 (4.99)
Ever had anal sex	
Yes	379 (94.51)
No	22 (5.49)
History of syphilis	
Yes	120 (29.93)
No	281 (70.07)
Duration of CA (year)	
< 1	284 (70.82)
≥ 1	117 (29.18)
CA therapy	
Yes	127 (31.67)
No	274 (68.33)
Duration of known HIV infection (year)	
< 3	271 (67.58)
≥ 3	130 (32.42)
HAART therapy (year)	
< 3	288 (71.82)
≥ 3	113 (28.18)
HIV-RNA (IU/ml)	
< 500	306 (76.31)
≥ 500	95 (23.69)
TH/TS ratio	
< 0.6	272 (67.83)
0.6–0.9	81 (20.20)
> 0.9	48 (11.97)
CD4^+^ count (cells/μl)	
< 200	54 (13.47)
200–500	201 (50.12)
> 500	146 (36.41)

CA, condyloma acuminata; HAART, highly active anti-retroviral therapy.

Descriptive baseline characteristics of the presence or absence of oncogenic HPV infection are shown in Table 2. There were significant differences in the participants’ age distribution (*p* = 0.01) and the median age (*p* = 0.04) between the two groups. Among the 302 participants with oncogenic HPV infections, the age band ≥35 accounted for the highest proportion, which is significantly higher than that of the uninfected group (30.79% vs. 14.14%, *p* = 0.01). The most notable differences between the two groups were that a higher percentage of participants with oncogenic HPV infections had a history of syphilis (35.43% vs. 13.13%, *p* < 0.01) compared with oncogenic HPV-uninfected participants, and the percentage of the duration of CA more than 1 year was also higher in participants with oncogenic HPV infections than those without them (30.79% vs. 24.24%, *p* < 0.01).

**Table 2:** Clinical and socio-demographic characteristics of the study population by oncogenic HPV infection status.

Characteristics	HPV16 or HPV18 infected*	Oncogenic HPV infected	Oncogenic HPV-uninfected	*p*#-value
	(*N* = 153)	(*N* = 302)	(*N* = 99)	
	*n* (%)	*n* (%)	*n* (%)	
Age group (year)				**0.01**
Median (IQR)	30 (26, 34)	30 (26, 37)	29 (26, 32)	**0.04**
≤ 26	32 (20.91)	68 (22.52)	25 (25.25)	
27–30	44 (28.75)	80 (26.49)	29 (29.29)	
31–34	39 (25.49)	61 (20.20)	31 (31.31)	
≥ 35	38 (24.83)	93 (30.79)	14 (14.14)	
Marriage				0.08
Yes	45 (29.41)	214 (70.86)	79 (79.80)	
No	108 (70.59)	88 (29.10)	20 (20.20)	
Men who had sex with men				0.20
Yes	145 (94.77)	286 (94.70)	95 (96.00)	
No	8 (5.23)	16 (5.30)	4 (4.00)	
Ever had anal sex				0.80
Yes	145 (94.77)	286 (94.70)	93 (93.90)	
No	8 (5.23)	16 (5.30)	6 (6.10)	
History of syphilis				**< 0.01**
Yes	59 (38.56)	107 (35.43)	13 (13.13)	
No	94 (61.44)	195 (64.57)	86 (86.87)	
Duration of CA (year)				**< 0.01**
< 1	110 (71.90)	209 (69.21)	75 (75.76)	
≥ 1	43 (28.10)	93 (30.79)	24 (24.24)	
CA therapy				0.09
Yes	48 (31.37)	103 (34.11)	24(24.24)	
No	105 (68.62)	199 (65.89)	75(75.76)	
Duration of known HIV infection (year)				0.40
< 3	104 (67.97)	208 (68.87)	63 (63.64)	
≥ 3	49 (32.03)	94 (31.13)	36 (36.36)	
HAART therapy (year)				0.15
< 3	116 (75.81)	223 (73.84)	65 (65.66)	
≥ 3	37 (24.18)	79 (26.16)	34 (34.34)	
HIV-RNA (IU/ml)				0.49
< 500	120 (78.43)	235 (77.81)	73 (73.74)	
≥ 500	33 (21.57)	67(22.19)	26 (26.26)	
TH/TS ratio				0.23
< 0.6	111 (72.55)	211 (69.87)	61 (61.62)	
0.6–0.9	25 (16.34)	59 (19.54)	22 (22.22)	
> 0.9	17 (11.11)	32 (10.60)	16 (16.16)	
**CD4**^**+**^count (cells/μl)				0.49
< 200	19 (12.42)	44 (14.57)	10 (10.10)	
200–500	76 (49.67)	151 (50.00)	50 (50.50)	
> 500	58 (37.91)	107 (35.43)	39 (39.39)	

CA, condyloma acuminata; HAART, highly active anti-retroviral therapy; IQR, interquartile range.

* Patients with HPV16 or HPV18 infection were included, regardless of single or multiple infection.

^#^ Oncogenic HPV-infected population compared to oncogenic HPV-uninfected population.

*p*-values less than 0.05 were shown in bold.

Beyond this, we found no statistical differences between the two groups concerning marriage, MSM, sexual behavior, CA therapy, duration of known HIV infection, HAART therapy, HIV-RNA, TH/TS ratio, or CD4^+^ count (*p* > 0.05).

In addition, 94.77% of the participants infected with HPV16 or HPV18 had anal sex, which was consistent with the proportion of MSM. Participants who had no history of syphilis, who were unmarried, and who had a duration of CA of less than 1 year were dominant in this group (Table 2).

### 3.3 Determinants of oncogenic HPV infection

Associations between oncogenic HPV infection and age, marital status, sexual behavior, orientation, history of syphilis, and CA or HIV were investigated in the oncogenic HPV-infected group. The results (ORs, 95% CIs, and *p*-values) of the univariate and multivariate analyses of potential influencing factors on oncogenic HPV infection in HIV-positive men with anal CA are shown in Table 3.

**Table 3:** Risk factors associated with oncogenic HPV infection in the HIV-positive patients with CA.

Covariate	Oncogenic HPV infected (*N* = 302)			
	COR (95% CI)	*p*-value	AOR (95% CI)	*p*-value
Age group (year)				
Continuous	1.04 (1.01–1.07)	**0.022**	1.03 (0.99–1.07)	0.11
≤ 26	1		1	
27–30	1.07 (0.59–1.95)	0.82	1.04 (0.57–1.90)	0.90
31–34	0.78 (0.42–1.48)	0.44	0.78 (0.41–1.48)	0.44
≥ 35	2.59 (1.33–5.30)	**0.01**	2.56 (1.20–5.70)	**0.02**
Marriage				
Yes	1.62 (0.95–2.87)	0.08	1.07 (0.57–2.05)	0.84
No	1		1	
Men had sex with men				
Yes	0.33 (0.05–1.16)	0.14	0.29 (0.05–1.06)	0.11
No	1		1	
Ever had anal sex				
Yes	0.29 (0.05–1.02)	0.09	0.29 (0.05–1.02)	0.09
No	1		1	
History of syphilis				
Yes	3.63 (1.99–7.09)	**< 0.01**	3.46 (1.90–6.79)	**< 0.01**
No	1		1	
Duration of CA (year)				
< 1	1		1	
≥ 1	0.64 (0.39–1.04)	0.07	0.62 (0.37–1.07)	0.08
CA therapy				
Yes	1.62 (0.98–2.76)	0.07	0.89 (0.52–1.54)	0.67
No	1		1	
Duration of known HIV infection (year)				
< 3	1		1	
≥ 3	1.21 (0.75–2.01)	0.44	5.62 (1.11–102.55)	0.09
HAART therapy (years)				
< 3	1		1	
≥ 3	0.99 (0.61–1.67)	0.98	0.20 (0.01–1.06)	0.13
HIV-RNA (IU/ml)				
< 500	1		1	
≥ 500	0.80 (0.48–1.37)	0.41	0.81 (0.48–1.40)	0.44
TH/TS ratio				
< 0.6	1		1	
0.6–0.9	0.77 (0.44–1.39)	0.38	0.83 (0.45–1.53)	0.53
> 0.9	0.58 (0.30–1.15)	0.15	0.62 (0.31–1.26)	0.17
CD4^+^ count (cells/μl)				
< 200	1		1	
200–500	0.70 (0.31–1.45)	0.36	0.75 (0.33–1.57)	0.47
> 500	0.61 (0.27–1.28)	0.21	0.73 (0.30–1.65)	0.46

CA, condyloma acuminata; HAART, highly active anti-retroviral therapy; COR, crude odds ratio; CI, confidence interval; AOR, adjusted odds ratio. *p*-values less than 0.05 were shown in bold.

Age as a continuous variable was entered into the model to determine the association of age with oncogenic HPV infection. Univariate analysis showed that the risk of oncogenic HPV infection increased with age at enrollment (COR: 1.04, 95% CI: 1.01–1.07, *p* = 0.022). However, the age of enrollment had no statistically significant effect on the oncogenic HPV infection after adjustment for covariates (AOR: 1.03, 95% CI: 0.99–1.07, *p* = 0.11).

It is worth noting that consistent results were yielded in univariate and multivariate analyses of the associations between age ≥ 35 years, history of syphilis, and oncogenic HPV infection. Participants who were ≥ 35 years (COR: 2.59, 95% CI: 1.33–5.30, *p* = 0.01) or had a history of syphilis (COR: 3.63, 95% CI: 1.99–7.09, *p* < 0.01) were more likely to harbor oncogenic HPV. Age ≥ 35 years (AOR: 2.56, 95% CI: 1.20–5.70, *p* = 0.02) and history of syphilis (AOR: 3.46, 95% CI: 1.90–6.79, *p* < 0.01) are independent risk factors for oncogenic HPV infection. Furthermore, marital status, sexual behavior, orientation, the initial diagnosis of HIV or CA, history of CA or anti-HIV treatment, and HIV-related parameters were not associated with oncogenic HPV infection.

The distribution of anal HPV infection types in HIV-positive patients with CA is displayed in Table 4. All 13 high-risk types were detected. The proportion of multiple infections was high as 96.51, and 6.73% and 2.99% of the participants were co-infected with bivalent and quadrivalent vaccine types, respectively. The most prevalent high-risk types were HPV52 (29.43%), HPV16 (28.93%), HPV59 (19.20%), and HPV18 (15.96%). Considering the more aggressive behavior of HPV16 or HPV18, the factors associated with HPV16 or HPV18 infection in HIV-positive CA participants were analyzed separately. Both univariate and multivariate analyses displayed a history of syphilis (AOR: 1.72, 95% CI: 1.08–2.73, *p* = 0.02) was the only risk factor associated with HPV16 or HPV18 infection (Table 5).

**Table 4:** Distribution of anal HPV genotypes in the HIV-positive patients with CA.

HPV genotype		Anal HPV prevalence (*N* = 401)	
		*n*	%
High-risk HPV	HPV16	11	**28.93**
	HPV18	64	**15.96**
	HPV31	45	11.22
	HPV33	60	14.96
	HPV35	19	4.74
	HPV39	48	11.97
	HPV45	51	12.92
	HPV51	59	14.71
	HPV52	118	**29.43**
	HPV56	32	7.98
	HPV58	59	14.71
	HPV59	77	**19.20**
	HPV68	57	14.21
Medium-risk HPV	HPV53	47	11.72
	HPV66	46	11.47
	HPV73	51	12.72
	HPV82	35	8.73
Low-risk HPV	HPV6	200	49.88
	HPV11	237	59.10
	HPV81	38	9.48
Multiple HPV types		387	96.51
Bivalent vaccine types	HPV 16/18	27*	6.73
Quadrivalent vaccine types	HPV 6/11/16/18	12#	2.99

^*^Number of participants infected with both HPV16 and HPV18.

^#^Number of participants co-infected with the quadrivalent vaccine type.

*p*-values less than 0.05 were shown in bold.

**Table 5:** Risk factors associated with HPV16 or HPV18 infection in the HIV-positive patients with CA.

Covariate	HPV16 or HPV18 infected (*N* = 153)			
	COR (95% CI)	*p*-value	AOR (95% CI)	*p*-value
Age group (year)				
Continuous	0.88 (0.71–1.08)	0.22	0.87 (0.70–1.01)	0.17
≤ 26	1		1	
27–30	1.00 (0.55–1.82)	1.00	1.01 (0.55–1.85)	0.98
31–34	0.92 (0.51–1.67)	0.78	0.94 (0.52–1.71)	0.84
≥ 35	0.64 (0.32–1.26)	0.20	0.60 (0.30–1.19)	0.15
Marriage				
Yes	1.24 (0.76–2.00)	0.39	1.25 (0.76–2.03)	0.37
No	1		1	
Men who had sex with men				
Yes	1.67 (0.60–5.91)	0.37	1.67 (0.60–5.94)	0.37
No	1		1	
Ever had anal sex				
Yes	1.89 (0.69–6.64)	0.26	1.89 (0.69–6.65)	0.26
No	1		1	
History of syphilis				
Yes	1.68 (1.06–2.65)	**0.03**	1.72 (1.08–2.73)	**0.02**
No	1		1	
Duration of CA (year)				
< 1	1		1	
≥ 1	1.20 (0.75–1.91)	0.45	1.20 (0.74–1.93)	0.45
CA therapy				
Yes	1.13 (0.72–1.79)	0.59	1.13 (0.71–1.80)	0.58
No	1		1	
Duration of known HIV infection (year)				
< 3	1		1	
≥ 3	1.04 (0.94–1.13)	0.45	1.02 (0.92–1.13)	0.8
HAART therapy (years)				
< 3	1		1	
≥ 3	1.47 (0.88–2.43)	0.15	1.27 (0.68–2.47)	0.47
HIV-RNA (IU/ml)				
< 500	1		1	
≥ 500	1.46 (0.51–5.23)	0.51	1.21 (0.39–4.56)	0.75
TH/TS ratio				
< 0.6	1		1	
0.6–0.9	0.71 (0.34–1.39)	0.34	0.67 (0.32–1.35)	0.28
> 0.9	0.57 (0.31–1.00)	0.06	0.54 (0.28–0.98)	0.05
CD4^+^ count (cells/μl)				
< 200	1		1	
200–500	1.30 (0.62–2.88)	0.50	1.61 (0.75–3.67)	0.24
> 500	1.53 (0.75–3.31)	0.26	1.59 (0.78–3.46)	0.22

CA, condyloma acuminata; HAART, highly active anti-retroviral therapy; COR, crude odds ratio; CI, confidence interval; AOR, adjusted odds ratio.

## 4 Discussion

The retrospective cohort study assessed the determinants of infection by oncogenic HPV in HIV-positive patients with CA in Shenzhen, Southeast China, hoping to provide insights into the screening and prevention of anal cancer. This study found that age ≥ 35 years and a history of syphilis were independent risk factors for oncogenic HPV infection among HIV-positive patients with anal CA. To the best of our knowledge, this is the first study to explore risk factors of oncogenic HPV infection in HIV-positive patients with anal CA.

We used exfoliated cells as samples to assess HPV infection in CA patients as in previous studies (30, 31). The sample was obtained by wiping CA lesions and the anal canal notwithstanding their inability to reveal the specific site of infection, but they reflected the HPV infection entire the anal region, not just in the anal canal. A sampling at different sites can promote the discovery of more HPV infections, which provides a basis for further exploration of the determinants of oncogenic infection.

Various risk factors associated with anal HPV infection have been reported in the HIV-positive population. The univariate analysis of this study displayed that oncogenic HPV infection increased with age at enrollment. In addition, age ≥ 35 years was an independent risk factor for oncogenic HPV infection, and this is not unrelated to the high proportion of patients with oncogenic HPV infection aged ≥35 years. Conversely, this might correlate with age-related prevalence and clearance of oncogenic HPV infection in the HIV-positive population. A collaborative pooled analysis of anal HPV epidemiology showed that HR-HPV prevalence in HIV-positive MSM increased from 58.3% at 15–18 years to 78.6% at 25–34 years (32). Geskus et al. reported that age had a statistically significant effect on HR-HPV clearance among HIV-positive MSM; the highest clearance rate of 12 types of HR-HPV was found under age 25, whereas subjects around age 30 seemed to have the lowest clearance rate (33). The higher prevalence and lower clearance of oncogenic HPV in older patients may partially explain the findings of this study. In contrast, similar studies have not found an association between age and HPV (or oncogenic HPV) infection in the HIV-positive population (34–36). This bias is likely due to the differences in study subjects enrolled. We focused on HIV-positive patients with CA, which limited the study population compared to other studies. From our findings, HIV-positive men with anal CA aged 35 years or older in Shenzhen can avoid HR-HPV infection and prevent the development of anal cancer if they benefit from HPV vaccination. The quadrivalent HPV vaccine has been reported to prevent the development of HPV-associated anal cancer in men aged 9–26 years (37), and it was not found to be effective in preventing anal HPV infection among HIV-positive patients aged 27 years or older (38). Therefore, HIV-positive patients ≤27 years of age with CA are more likely to benefit from vaccination, given the longer protective duration of the HPV vaccine.

In multivariate analysis, we found that syphilis history is the strongest risk factor associated with carcinogenic HPV infection in HIV-positive patients with CA, and a similar conclusion has been reported. Nagata et al. found that having ≥2 sexually transmitted infections (including syphilis) was independently associated with carcinogenic HPV infection among Japanese HIV-infected patients (39). As one of the most common sexually transmitted infections (40), syphilis demonstrated a high incidence of co-infection with HPV (41). It has been reported that secondary syphilis patients exhibited a significant decrease in total circulating NK-cell populations, and treponema pallidum continues to spread into the bone marrow, which may affect the development of myeloid and lymphoid progenitors of NK-cells during secondary syphilis (42), while the NK cells play an important role in resisting virus invasion. The increased incidence of HPV infection and HPV-associated cancer incidence has been discovered in individuals with various functional NK cell deficiencies (43, 44), suggesting that immune factors are responsible for the increased risk of HPV infection in syphilis patients. Notably, sexually active individuals and individuals who have unprotected sex individuals are at high risk for syphilis and HPV infection (45), this makes it impossible to determine the specific infection process of the two pathogens in the study population. Although we could not determine whether the participants were infected with syphilis or HPV first, a history of syphilis in HIV-positive patients with CA is still worthy of attention. In addition, as a retrospective study, stage and specific treatment situations of syphilis were not available, which prevented us from exploring the effect of syphilis treatment history on HR-HPV infection. Early standardized treatment and intervention after diagnosis of sexually acquired syphilis may make HIV-positive CA patients less susceptible to HR-HPV anal infection, which deserves further study.

It should be mentioned that no association between HIV-specific factors and carcinogenic HPV infection was found in the study. Previous studies on the effect of HIV-specific factors on carcinogenic HPV infection were inconsistent in HIV-positive individuals. A longitudinal study in Italy reported that CD4^+^ count and CDC stage C diagnosis were independently associated with anal HR-HPV infection (46). Low CD4 count (< 350 cells/mm^3^) and previous chlamydia infection were associated with an increased risk of carcinogenic HPV infection among HIV-infected MSM in Northern California (47). For HIV-positive individuals in Southeast Asia, baseline CD4 count <350 cells/mm^3^ showed a trend toward increased risk of any anal HR-HPV persistence, while antiretroviral therapy use and plasma HIV-RNA had no association with any anal HR-HPV persistence (34), whereas, in the same region, another investigation revealed that CD4^+^ count, HIV-RNA, or use of combination antiretroviral therapy were not significantly associated with the prevalence of anal HR-HPV types in HIV-positive MSM. Differences in population characteristics due to different inclusion criteria may be the main reason for biased results. Multiple studies have reported a correlation between HIV parameters and anal dysplasia (48, 49). The relative risk for anal squamous intraepithelial lesions among HIV-positive men increased with lower CD4 levels (49). In this study, participants with a diagnosis of anal precancer were rejected at enrollment, which is equivalent to excluding participants with relatively poor HIV disease status. Only 14.57% of the participants had CD4^+^ count <200 cells/ul among the carcinogenic HPV-infected participants, which confirmed this possibility to a certain extent.

This study was not without limitations. It was a single-center survey, and the participants in our study might not represent all HPV-positive patients in Shenzhen city. As a retrospective study, we were unable to obtain comprehensive data on sociodemographics, sexual intercourse, and sexually transmitted infection, as well as factors such as smoking (50), ethnicity (36), number of sexual partners, frequency of intercourse (51), and chlamydia infection (47), which had been reported to be associated with HPV infection. In future, a multicenter prospective survey based on larger populations is required to generalize the findings of Shenzhen HIV-positive patients with CA.

## 5 Conclusion

In China, HIV-positive patients with CA often do not undergo HPV testing in their daily medical routine. On the premise of not increasing the burden of treatment for patients, reducing the risk of oncogenic HPV infection in high-risk populations may be the key to preventing the development of anal cancer. The retrospective cohort study found HIV-positive CA patients aged ≥35 years or with a history of syphilis were more likely to harbor oncogenic HPV, and the history of syphilis was also an independent risk factor associated with HPV16 or HPV18 infection. These results provide a strategy reference for the prevention of oncogenic HPV infection, which is beneficial to reduce the cost of anal cancer prevention for high-risk groups and expand more perspectives for follow-up research in this field. Moreover, it provided a feasible theoretical basis for establishing anal cancer prevention strategies in China and other developing countries.

## Data availability statement

The datasets presented in this article are not readily available because the dataset contains some private information of participants and we are not authorized to share the clinical data of the patients from the hospital. Requests to access the datasets should be directed to weilanlan@mail.sustech.edu.cn.

## Ethics statement

The studies involving humans were approved by Medical Ethic Committee in the Third People’s Hospital of Shenzhen. The studies were conducted in accordance with the local legislation and institutional requirements. The participants provided their written informed consent to participate in this study.

## Author contributions

LW conceived and designed the study. RY, XZ, and DL were involved in data collection and analysis. JL, WC, and FW drafted the manuscript. All authors contributed to the article and approved the submitted version.
